# Natural and Experimental Infection of *Caenorhabditis* Nematodes by Novel Viruses Related to Nodaviruses

**DOI:** 10.1371/journal.pbio.1000586

**Published:** 2011-01-25

**Authors:** Marie-Anne Félix, Alyson Ashe, Joséphine Piffaretti, Guang Wu, Isabelle Nuez, Tony Bélicard, Yanfang Jiang, Guoyan Zhao, Carl J. Franz, Leonard D. Goldstein, Mabel Sanroman, Eric A. Miska, David Wang

**Affiliations:** 1 Institut Jacques Monod, CNRS-University of Paris-Diderot, Paris, France; 2 Gurdon Institute, University of Cambridge, Cambridge, United Kingdom; 3 Departments of Molecular Microbiology and Pathology & Immunology, Washington University in St. Louis School of Medicine, St. Louis, Missouri, United States of America; Oxford University, United Kingdom

## Abstract

An ideal model system to study antiviral immunity and host-pathogen co-evolution would combine a genetically tractable small animal with a virus capable of naturally infecting the host organism. The use of *C. elegans* as a model to define host-viral interactions has been limited by the lack of viruses known to infect nematodes. From wild isolates of *C. elegans* and *C. briggsae* with unusual morphological phenotypes in intestinal cells, we identified two novel RNA viruses distantly related to known nodaviruses, one infecting specifically *C. elegans* (Orsay virus), the other *C. briggsae* (Santeuil virus). Bleaching of embryos cured infected cultures demonstrating that the viruses are neither stably integrated in the host genome nor transmitted vertically. 0.2 µm filtrates of the infected cultures could infect cured animals. Infected animals continuously maintained viral infection for 6 mo (∼50 generations), demonstrating that natural cycles of horizontal virus transmission were faithfully recapitulated in laboratory culture. In addition to infecting the natural *C. elegans* isolate, Orsay virus readily infected laboratory *C. elegans* mutants defective in RNAi and yielded higher levels of viral RNA and infection symptoms as compared to infection of the corresponding wild-type N2 strain. These results demonstrated a clear role for RNAi in the defense against this virus. Furthermore, different wild *C. elegans* isolates displayed differential susceptibility to infection by Orsay virus, thereby affording genetic approaches to defining antiviral loci. This discovery establishes a bona fide viral infection system to explore the natural ecology of nematodes, host-pathogen co-evolution, the evolution of small RNA responses, and innate antiviral mechanisms.

## Abstract 2

Novel viruses have been discovered in wild *Caenorahbditis* nematode isolates and can now be used to explore host antiviral pathways, nematode ecology, and host-pathogen co-evolution.

## Author Summary

The nematode *C. elegans* is a robust model organism that is broadly used in biology. It also has great potential for the study of host-microbe interactions, as it is possible to systematically knockout almost every gene in high-throughput fashion to examine the potential role of each gene in infection. While *C. elegans* has been successfully applied to the study of bacterial infections, only limited studies of antiviral responses have been possible since no virus capable of infecting any *Caenorhabditis* nematode in laboratory culture has previously been described. Here we report the discovery of natural viruses infecting wild isolates of *C. elegans* and its relative *C. briggsae*. These novel viruses are most closely related to the ssRNA nodaviruses, but have larger genomes than other described nodaviruses and clearly represent a new taxon of virus. We were able to use these viruses to infect a variety of laboratory nematode strains. We show that mutant worms defective in the RNA interference pathway, an antiviral system known to operate in a number of organisms, accumulate more viral RNA than wild type strains. The discovery of these viruses will enable further studies of host-virus interactions in *C. elegans* and the identification of other host mechanisms that counter viral infection.

## Introduction

Model organisms such as *D. melanogaster* [1],[2] and *C. elegans* [3],[4] have been increasingly used in recent years to examine features of the host immune system and host-pathogen co-evolution mechanisms, due to the genetic tractability and ease of manipulation of these organisms. A prerequisite to fully exploit such models is the identification of an appropriate microbe capable of naturally infecting the host organism. Analysis in *C. elegans* of bacterial pathogens such as *Pseudomonas*, *Salmonella*, or *Serratia* has been highly fruitful, in some instances revealing the existence of innate immune pathways in *C. elegans* that are also conserved in vertebrates [3]. The recent report of natural infections of *C. elegans* intestinal cells by microsporidia makes it a promising model for microsporidia biology [5]. Efforts to use *C. elegans* to understand anti-viral innate immunity, however, have been hampered by the lack of a natural virus competent to infect and replicate in *C. elegans*.

In the absence of a natural virus infection system, some efforts to define virus-host responses in *C. elegans* have been pursued using artificial methods of introducing viruses or partial virus genomes into animals [6],[7]. For example, the use of a transgenic Flock House virus RNA1 genome segment has clearly established a role for RNAi in counteracting replication of Flock House virus RNA [7] and has defined genes essential for the RNAi response [8]. However, this experimental system can only examine replication of the viral RNA and is fundamentally unable to address the host response to other critical aspects of the virus life cycle such as virus entry, virion assembly, or egress. The ability of a host to target steps other than genome replication to control viral infections is highlighted by recent discoveries such as the identification of tetherin, which plays a critical role at the stage of viral egress by blocking the release of fully assembled HIV virions from infected human cells [9]. Furthermore, the artificial systems used to date for analysis of virus-nematode interactions cannot be used to examine transmission dynamics of virus infection. These limitations underscore the need to establish an authentic viral infection and replication system in nematodes.

Natural populations of *C. elegans* have proven hard to find until recent years. The identification of *C. elegans* habitats and the development of simple isolation methods (MAF, unpublished) [10] has now enabled extensive collection of natural isolates of *C. elegans*. Here we report the discovery of natural populations of *C. elegans* and of its close relative *C. briggsae* that display abnormal morphologies of intestinal cells. These abnormal phenotypes can be maintained in permanent culture for several months, without detectable microsporidial or bacterial infection. We show that these populations are infected by two distinct viruses, one specific for *C. elegans* (Orsay virus), one for *C. briggsae* (Santeuil virus). These viruses resemble viruses in the *Nodaviridae* family, with a small, bipartite, RNA(+sense) genome. Infection by each virus is transmitted horizontally. In both nematode species, we find intraspecific variation in sensitivity to the species-specific virus. We further show that infected worms mount a small RNA response and that RNAi mechanisms act as antiviral immunity in nematodes. Finally, we demonstrate that the *C. elegans* isolate from which Orsay virus was isolated is incapable of mounting an effective RNAi response in somatic cells. We thus find natural variation in host antiviral defenses. Critically, these results establish the first experimental viral infection system in *C. elegans* suitable for probing all facets of the host antiviral response.

## Results

### Natural Viral Infections of *C. briggsae* and *C. elegans*

**Figure 1:**
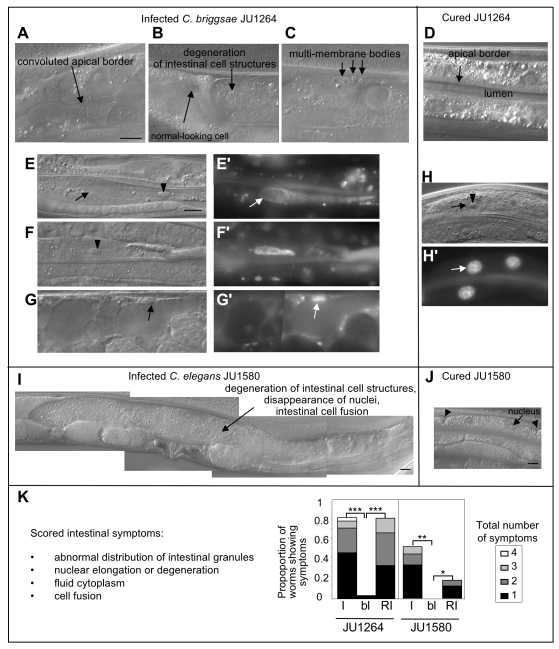
Intestinal cell infection phenotypes in wild *Caenorhabditis* isolates. (A–H) *C. briggsae* JU1264 and (I,J) *C. elegans* JU1580 observed by Nomarski microscopy. (A–C, E–G, I) Infected adult hermaphrodites from the original cultures, with the diverse infection symptoms: convoluted apical intestinal border (A), degeneration of intestinal cell structures and liquefaction of the cytoplasm (B, G, I), presence of multi-membrane bodies (C). The animals in (E–H) were also observed in the fluorescence microscope after live Hoechst 33342 staining of the nuclei, showing the elongation and degeneration of nuclei (E′–H′). In (E), the nucleus and nucleolus are abnormally elongated. In (F), the nuclear membrane is no longer visible by Nomarski optics. In (G), the cell cytoplasmic structures are highly abnormal (apparent vacuolisation) and the nucleus is very reduced in size. In (E–H′), arrows denote nuclei and arrowheads nucleoli. The infected animal in (I) displays an abnormally large intestinal cell that is probably the result of cell fusions, with degeneration of cellular structures including nuclei. (D, H, J) Uninfected (bleached) adults. Arrowheads in (J) indicate antero-posterior boundaries between intestinal cells, each of which generally contains two nuclei. Bars: 10 µm. (K) Proportion of worms showing the indicated cumulative number of morphological infection symptoms in at least one intestinal cell, in the original wild isolate (I), after bleaching (bl) and after re-infection by a 0.2 µM filtrate (RI). Note that not all symptoms shown in (A–I) were scored, because some are difficult to score or may also occur in healthy animals. The animals were scored 4 d after re-infection for *C. briggsae* JU1264, and 7 d after re-infection for *C. elegans* JU1580, at 23°C. The symptoms are similar in both species, and generally more frequent in JU1264. *** *p* value on number of worms showing infection symptoms <7.10^−11^, Fisher's exact test; ** *p* value<3.10^−6^; * *p* value<3.10^−2^.

**Figure 2:**
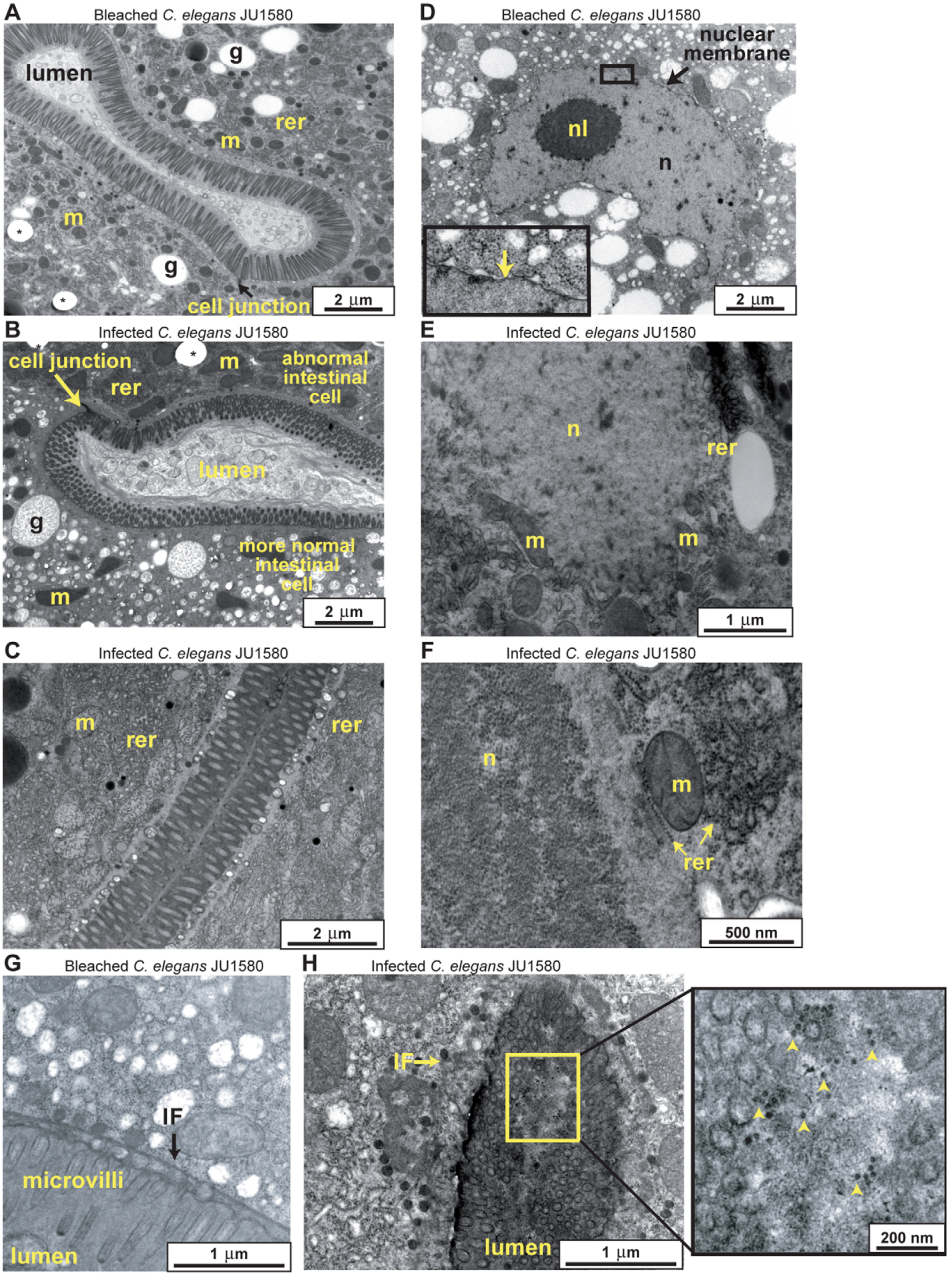
Transmission electron micrographs of intestinal cells of *C. elegans* JU1580 adult hermaphrodites. (A,D,G) Bleached animals. (B–C, E–F, H) Naturally infected animals. (A–C) The infection provokes a reorganization of cytoplasmic structures, most visibly the loss of intestinal lipid storage granules (g). The cytoplasm of infected intestinal cells mostly contains rough endoplasmic reticulum (rer) and mitochondria (m). * hole in the resin used for inclusion in electron microscopy. (D–F) A nucleus in a non-infected animal is surrounded by a nuclear membrane (see inset in D), whereas the nuclear membrane disappears upon infection (E–F). Absence or incomplete nuclear membrane was observed repeatedly in infected animals, while the nuclear membrane could be observed on bleached animals (using both fixation methods). The nuclear material (n) in (F) may represent nucleolar material and at lower magnification (not shown) matches the shape of elongated nucleoli as observed by Nomarski optics (Figure 1E–G). The rough endoplasmic reticulum (rer) on the left of the mitochondrion (m) in (F) may be a remnant of the nuclear envelope. (G–H) The infection may result in disorganization of the intermediate filament (IF) network normally located below the apical plasma membrane. On the right of (H) is shown a higher magnification of the intestinal lumen, showing putative viral particles (arrowheads). The animals were fixed using high-pressure freezing (A–C, E–F) or conventional fixation (D, G, H).

From surveys of wild nematodes from rotting fruit in different regions of France, multiple *Caenorhabditis* strains were isolated that displayed a similar unusual morphology of the intestinal cells and no visible pathogen by optical microscopy. Intestinal cell structures such as storage granules disappeared (Figures 1A–J, 2A–C) and the cytoplasm lost viscosity and became fluid (Figure 1B,I), moving extensively during movement of the animal. The intestinal apical border showed extensive convolutions and intermediate filament disorganization (Figures 1A, 2H, as described in some intermediate filament mutants, [11]). Multi-membrane structures were sometimes apparent in the cytoplasm (Figure 1C). Elongation of nuclei and nucleoli, and nuclear degeneration, were observed using Nomarski optics, live Hoechst 33342 staining, and electron microscopy (Figures 1E–H, 2D–F). Finally, some intestinal cells fused together (Figure 1I). This suite of symptoms was first noticed during sampling of *C. briggsae*. Indeed, more individuals appeared affected in *C. briggsae* than in *C. elegans* cultures, and to a greater extent (Figure 1K).

One representative, stably infected, strain of each nematode species, *C. elegans* JU1580 (isolated from a rotting apple in Orsay, France) and *C. briggsae* JU1264 (isolated from a snail on a rotting grape in Santeuil, France), were selected for detailed analysis. Bleaching of adult animals resulted in phenotype-free progeny from both strains, demonstrating that the phenotype was not vertically transmitted (embryos are resistant to the bleaching treatment) (Figure 1K). Addition of dead infected animals, or homogenates from infected animals after filtration through 0.2 µm filters, to plates containing previously bleached animals recapitulated the morphological phenotype, raising the possibility that a virus might play a role in inducing the morphological phenotype (Figure 1K). We found that the infectious agent could be passed on horizontally through live animals by incubating GFP-labeled animals (strain JU1894, Table 1) with 10 non-GFP-infected worms (JU1580), checking that the latter did not die before removing them 24 h later. The GFP-labeled culture displayed the intestinal symptoms after a week. One possibility is that the intestinal infectious agent is shed from the intestine through the rectum and may enter the next animal during feeding.

**Table 1:** Strain list.

Strain	Genotype
JU1264	*C. briggsae* wild isolate, Santeuil, France
JU1580	*C. elegans* wild isolate, Orsay, France
AF16	*C. briggsae* wild reference isolate, India
N2	*C. elegans* wild reference isolate, England
CB4856	*C. elegans* wild isolate, Hawaii
AB1	*C. elegans* wild isolate, Australia
PB303	*C. elegans* wild isolate, USA
PB306	*C. elegans* wild isolate, USA
JU258	*C. elegans* wild isolate, Madeira
PS2025	*C. elegans* wild isolate, California, USA
JU1894	*mfEx50*[*let858*::GFP, *myo*-*2*::DsRed] in JU1580 background
JU1895	*mfEx51*[*let858*::GFP, *myo*-*2*::DsRed] in N2 background
WM27	*rde-1(ne219)* V
WM29	*rde-2(ne221)* I
WM49	*rde-4(ne301)* III
NL936	*unc-32(e189) mut-7(pk204)* III

In support of the hypothesis that these wild *Caenorhabditis* were infected by a virus, small virus-like particles of approximately 20 nm diameter were visible by electron microscopy of the intestinal cells (Figures 2H, S1). Such particles were not observed in bleached animals, nor in *C. elegans* animals infected by bacteria, which showed a strong reduction of intestinal cell volume (strain JU1409, unpublished data).

While a clear morphological phenotype was visible by microscopy, infection did not cause a dramatic decrease in adult longevity (unpublished data), nor a change in brood size (Figure S2A,B). However, progeny production was significantly slowed down during adulthood, most clearly in the infected *C. briggsae* JU1264 isolate compared to the uninfected control (Figure S2D).

### Molecular Identification of Two Divergent Viruses

**Figure 3:**
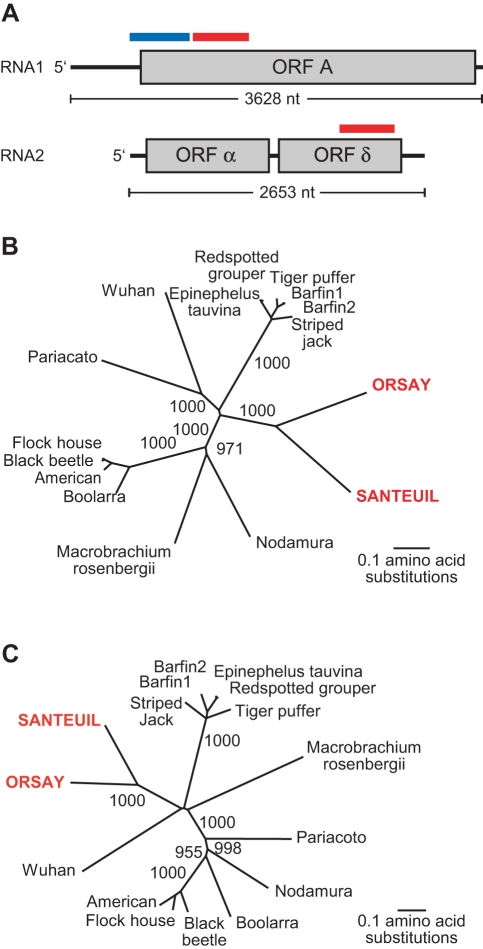
Genomic organization and phylogenetic analysis of novel viruses. (A) Schematic of genomic organization of Santeuil virus. Predicted open reading frames are displayed in gray boxes. Red bar indicates sequence used to generate double-stranded DNA probes for Northern blotting. Blue bar indicates sequence used to generate single-stranded riboprobes. (B) Neighbor-joining phylogenetic analysis of the predicted RNA-dependent RNA polymerases encoded by the RNA1 segments. (C) Neighbor-joining phylogenetic analysis of the predicted capsid proteins encoded by the RNA2 segments.

An unbiased high-throughput pyrosequencing approach was used to determine whether any known or novel viruses were present in the animals. From JU1264, 28 unique sequence reads were identified initially that shared 30%–48% amino acid sequence identity to known viruses in the family *Nodaviridae*. Nodaviruses are bipartite positive strand RNA viruses. The RNA1 segment of all previously described nodaviruses is ∼3.1 kb and encodes ORF A, the viral RNA-dependent RNA polymerase. Some nodaviruses also encode ORFs B1/B2 at the 3′ end of the RNA1 segment. The B1 protein is of unknown function while the B2 protein is able to inhibit RNAi [12]. The RNA2 segment of all previously described nodaviruses is ∼1.4 kb and possesses a single ORF encoding the viral capsid protein. Assembly of the initial JU1264 pyrosequencing reads followed by additional pyrosequencing, RT-PCR, 5′ RACE, and 3′ RACE yielded two final contigs, which were confirmed by sequencing of overlapping RT-PCR amplicons. The two contigs corresponded to the RNA1 and RNA2 segments of a novel virus. The first contig (3,628 nt) encoded a predicted open reading frame of 982 amino acids that shared 26%–27% amino acid identity to the RNA-dependent RNA polymerase of multiple known nodaviruses by BLAST alignment. All known nodavirus B2 proteins overlap with the C-terminus of the RNA-dependent RNA polymerase and are encoded in the +1 frame relative to the polymerase. No open reading frame with these properties was predicted in the 3′ end of the RNA1 segment. The second contig of 2,653 nt, which was presumed to be the near-complete RNA2 segment, encoded at its 5′ end a predicted protein with ∼30% identity to known nodavirus capsid proteins (Figure 3A). This contig was ∼1 kb larger than the RNA2 segment of all previously described nodaviruses and appeared to encode a second ORF of 332 amino acids at the 3′ end. This second predicted ORF, named ORF δ, had no significant BLAST similarity to any sequence in Genbank.

Pyrosequencing of JU1580 demonstrated the presence of a second distinct virus that shared the same general genomic organization as the virus detected in JU1264. Partial genome sequences of 2,680 nucleotides of the RNA1 segment and 2,362 nucleotides of the RNA2 segment were obtained and confirmed by RT-PCR. The putative RNA-dependent RNA polymerases of the two viruses shared 44% amino acid identity by BLAST analysis. Like the virus in JU1264, the virus in JU1580 was predicted to encode a capsid protein at the 5′ end of the RNA2 segment as well as a second ORF in the 3′ half of the RNA2 segment. The ORF δ encoded proteins from the two viruses shared 37% amino acid identity when compared using BLAST. Thus, the genomic organization of these two viruses, while sharing substantial commonality with known nodaviruses, also displayed novel genomic features. Phylogenetic analysis of the predicted RNA polymerase and capsid proteins demonstrated that the virus sequences in JU1580 and JU1264 were highly divergent from all previously described nodaviruses and most closely related to each other (Figure 3B,C). We propose that these sequences represent two novel virus species and have tentatively named them Santeuil virus (from JU1264) and Orsay virus (from JU1580).

### Viral Detection and Confirmation of Viral Infection

**Figure 4:**
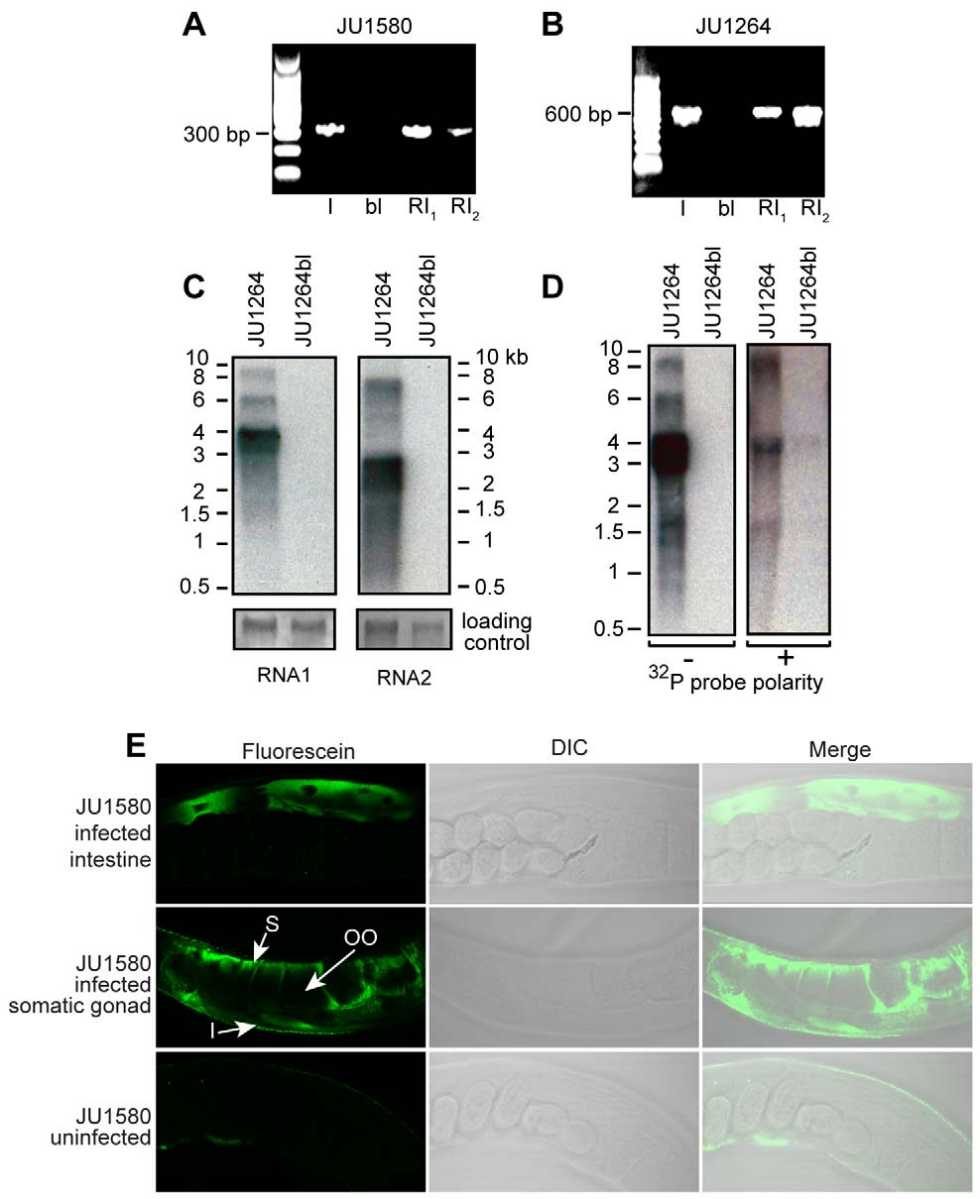
Molecular evidence of viral infection. (A) RT-PCR detection of the Orsay virus in the original JU1580 wild isolate (I), after bleaching (bl) and after re-infection by a 0.2 µM filtrate after 7 d (RI_1_) and 3 wk (RI_2_) of culture at 23°C. (B) RT-PCR detection of the Santeuil virus in the original wild isolate (I), after bleaching (bl) and after re-infection by a 0.2 µM filtrate after 4 d (RI_1_) and 4 wk (RI_2_) at 23°C. (C) Northern blots of Santeuil virus RNA1 and RNA2 segments hybridized using a double-stranded DNA probe. (D) Northern blots of Santeuil virus RNA1 segment using + and − sense riboprobes. (E) RNA FISH with a probe targeting Orsay virus RNA1 segment. Representative JU1580bl animals following infection by Orsay virus (top and middle rows) or uninfected (bottom row). S corresponds to ovary sheath cells, OO is an oocyte, and I is an intestinal cell.

RT-PCR assays were used to analyze RNA extracted from JU1580, JU1264, their corresponding bleached control strains JU1580bl and JU1264bl, and the same strains following reinfection with viral filtrates. Orsay virus RNA could be detected by RT-PCR in the original JU1580 culture, disappeared in the bleached strains, and stably reappeared following re-infection with the corresponding viral filtrate (Figure 4A). The same pattern applied for the Santeuil virus and JU1264 animals (Figure 4B). JU1580 and JU1264 cultures continuously propagated for 6 mo by transferring a piece of agar (approx. 0.1 cm^3^) to the next plate twice a week continued to yield positive RT-PCR results (unpublished data).

Northern blotting confirmed the presence of Orsay and Santeuil virus RNA sequences in the infected animals. Hybridization with a DNA probe targeting the RNA1 segment of Santeuil virus yielded multiple bands in JU1264 animals but not in the corresponding bleached control strain. The strongest band detected migrated between 3.5 and 4 kb consistent with the 3,628 nt sequence we generated for the putative complete RNA1 segment (Figure 4C). Multiple higher molecular weight bands were also detected that may represent multimeric forms of the viral genomic RNAs, which have previously been described for some nodaviruses [13],[14]. Northern blotting with a probe targeting the RNA2 segment (Figure 4C) yielded a major band that migrated at ∼2.5 kb as well as fainter, higher molecular weight bands. Similar patterns were seen for both segments of Orsay virus (unpublished data).

To demonstrate virus replication in the infected animals, we performed Northern blotting using strand-specific riboprobes. For positive sense RNA viruses like nodaviruses, the negative sense RNA is only synthesized during active viral replication. It is not packaged in virions and typically exists in much lower quantities than the positive strand. Robust levels of the positive strand of the Santeuil virus RNA1 segment were detected (Figure 4D). Northern blotting with a riboprobe designed to hybridize to the negative sense strand detected a band of ∼3.5 kb as well as higher molecular weight bands and a lower band of ∼1.5 kb (∼30-fold longer exposure than the positive sense blot; Figure 4D). While the precise nature of the high and low molecular weight species remains to be defined, the presence of multiple RNA species of negative sense polarity in JU1264 animals demonstrates bona fide replication of Santeuil virus in JU1264.

In order to determine the localization of Orsay viral RNA in infected animals, we performed RNA fluorescent in situ hybridization (FISH) using a probe complementary to the positive sense RNA1 segment of Orsay virus. Viral RNA was robustly detected in intestinal cells of JU1580bl animals infected 4 d previously with Orsay viral filtrate (Figure 4E, top panels). Interestingly, some animals also showed localization of viral RNA in the somatic gonad (Figure 4E, middle panels). JU1580bl animals not treated with the viral filtrate displayed no fluorescent signal (Figure 4E, bottom panels).

### High Specificity of Infection by Orsay and Santeuil Nodaviruses

**Figure 5:**
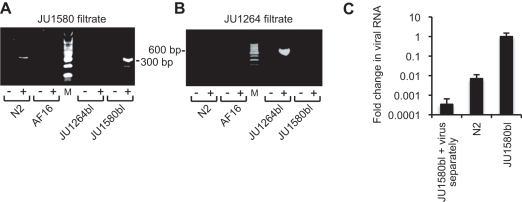
Specificity of infection by the Orsay and Santeuil viruses. (A) Specificity of infection by the Orsay virus. Each *Caenorhabditis* strain (name indicated below the gel) was mock-infected (−) or infected with a virus filtrate (+). RT-PCR on cultures after 7 d at 23°C. See Figure S3 for corresponding morphological symptom scoring. (B) Specificity of infection by the Santeuil virus. RT-PCR results after 4 d at 23°C. (C) Quantitative variation in viral replication N2 versus JU1580. N2 and JU1580 were tested by qRT-PCR for infection with Orsay virus extract (*n* = 10 independent replicates for each strain). By conventional RT-PCR assay, Orsay virus infection of N2 yielded positive bands in 3 out of 10 replicate infections whereas 7 out of 10 replicate infections of JU1580bl were positive in these conditions. Control RNA (*n* = 6) was extracted from JU1580bl animals grown in parallel without virus filtrate, and to which filtrate was added at the time of sample collection. RNA levels were normalized to *ama-1* and shown as average fold-change relative to JU1580bl. Error bars represent SEM.

We tested whether the Orsay and Santeuil viruses could cross-infect the cured wild isolate of the other *Caenorhabditis* species, as well as the reference laboratory strains of *C. elegans* and *C. briggsae*. The Orsay and Santeuil viruses could only infect strains of *C. elegans* and *C. briggsae*, respectively (Figure 5A,B and Figure S3). Furthermore, each virus showed intraspecific specificity of infection. Indeed, we could not detect any replication of the Santeuil virus in *C. briggsae* AF16. The N2 laboratory *C. elegans* strain, while infectable by Orsay virus, appeared to be more resistant to viral infection than JU1580bl. Quantitative RT-PCR demonstrated that viral RNA accumulated in the N2 strain at levels above background but 50–100-fold lower than in JU1580bl (Figure 5C).

### Small RNA Response upon Infection

**Figure 6:**
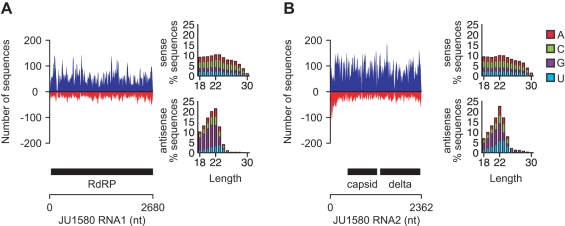
Small RNAs produced upon viral infection. Number of unique sequences obtained by Illumina/Solexa high-throughput sequencing of a 5′-independent small RNA library from JU1580 matching a given position in the Orsay virus segment RNA1 (A) or RNA2 (B). The number of sequences in sense and antisense orientation are shown on the positive (blue) and negative (red) *y*-axis, respectively. Only sequences with a perfect and unambiguous match to the virus genome were considered. The location of virus protein-coding genes is indicated below each graph as black bars and the RNA genome as a line. Features of sense and antisense sequences (length and identity of first nucleotide) are shown to the right of each graph.

One key defense mechanism of plants and animals against RNA viruses is the small RNA response [15]. We therefore determined by deep sequencing of small RNAs whether the infected animals produced small RNAs in response to viral infection. We generated small RNA libraries from mixed-stage JU1580 animals infected with the Orsay virus and from the bleached control strain and analyzed them using Illumina/Solexa high-throughput sequencing. These libraries represent small RNAs of 18–30 nucleotides in length independent of their 5′ termini. Small RNAs from infected JU1580 animals that mapped to viral RNA1 or RNA2 and had no match to the *C. elegans* genome are shown in Figure 6A and 6B, respectively. Of a total of 1,149,633 unambiguously mapped unique sequences, almost 2% (21,392) mapped to the two RNA segments of Orsay virus. Such RNAs were virtually absent from a library generated from bleached JU1580 animals (<0.001%) (unpublished data). Small RNAs that corresponded to the sense strand of the viral RNAs had a broad length distribution and no 5′ nucleotide preference. These sense small RNAs might represent Dicer cleavage products or other viral RNA degradation intermediates. In contrast, most antisense small RNAs were 22 nt long and showed a bias for guanidine as the first base (Figure 6A,B). This signature is reminiscent of a class of secondary RNAs named 22G RNAs that are thought to be downstream effectors of exogenous and endogenous small RNA pathways [16]–[18]. Such RNAs are not associated with transgenes expressed in the soma of *C. elegans* from extrachromosomal arrays [17] nor generally a feature associated with active transcription of endogenous genes [16]–[18]. These data suggest that JU1580 animals raise a small RNA response to viral infection. We also detected small RNAs of both sense and antisense polarity that mapped to the Santeuil virus genome in the JU1264 wild *C. briggsae* isolate but not in bleached animals (unpublished data).

### RNAi Competency of the Host Is an Antiviral Defense

**Figure 7:**
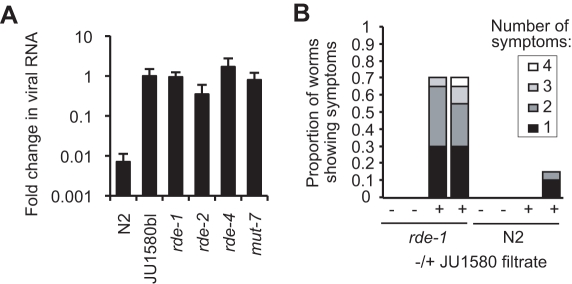
RNAi-deficient mutants of *C. elegans* can be infected by the Orsay virus. (A) JU1580bl, N2, *rde-1(ne219)* (*n* = 10 independent replicates each), *rde-2(ne221)*, *rde-4(ne301)*, and *mut-7(pk204)* (*n* = 5 independent replicates each) were tested by qRT-PCR for infection with Orsay virus extract. RNA levels were normalized to *ama-1* and shown as average fold-change relative to JU1580bl. Error bars represent SEM. Same results as displayed in Figure 5C for N2 and JU1580. (B) Scoring of symptoms in two independent replicates of infection of *rde-1* mutant and wild-type N2 animals by the Orsay virus filtrate, after 4 d.

As viral infection appears to invoke a small RNA response in JU1580 animals, we next tested if mutations in small RNA pathways could affect replication of the Orsay virus. Orsay virus infection of the N2 reference strain was reduced compared to JU1580, as assayed by viral RNA qRT-PCR (Figure 5C) and infection symptoms (Figures S3A and 7B). Mutation of the *rde-1* gene—which encodes an Argonaute protein required for the initiation of exogenous RNAi [19]—in the N2 background increased viral RNA abundance and morphological symptoms to levels comparable to JU1580 using both assays (Figure 7A,B). The infected *rde-1* strain produced infectious viral particles, as reinfection of the cured JU1580 strain by filtrates of infected *rde-1* animals yielded positive RT-PCR results (unpublished data). In addition, mutation of other exogenous RNAi pathway genes including *rde-2*, *rde-4*, and *mut-7* (Table 1) also led to increased viral RNA accumulation as determined by quantitative RT-PCR (Figure 7A). We thus conclude that RNAi mechanisms provide antiviral immunity to *C. elegans* and that Orsay virus infection of mutant animals can be used to define genes important for antiviral defense.

### Natural Variation in Somatic RNAi Efficiency in *C. elegans*

**Figure 8:**
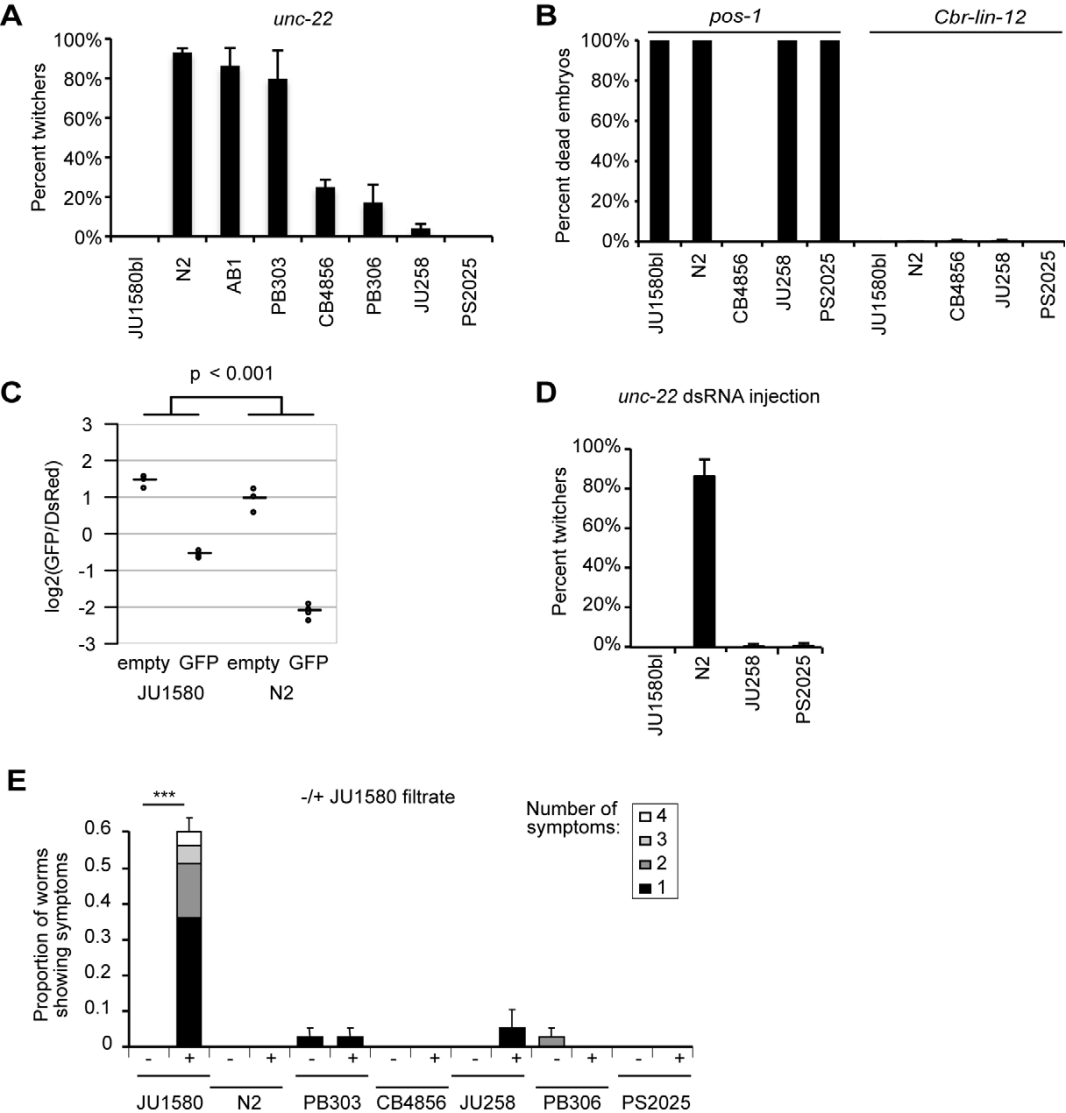
Natural variation in somatic RNAi efficacy in *C. elegans*. (A) Somatic RNAi was tested using bacteria expressing dsRNA specific for the *unc-22* gene (acting in muscle; [37]). The percentage of animals with the corresponding twitcher phenotype is shown for different *C. elegans* wild isolates (representative of the species' diversity; [38]). Bar: standard error over four replicate plates. (B) Germline RNAi was tested by feeding the animals with bacteria expressing dsRNA specific for the *pos-1* gene. The percentage of animals with the corresponding embryonic-lethal phenotype is shown for five wild genetic backgrounds of *C. elegans*. *Cbr-lin-12* RNAi is a negative control. Bar: standard error over six replicate plates (too small to be seen). *n*>450 observed individuals for each treatment. (C) Somatic RNAi was tested using bacteria expressing dsRNA specific for GFP. Each point corresponds to the median log_2_(GFP/DsRed) intensity ratio from one flow cytometry run of strains carrying the *let-858::GFP* transgene in the JU1580 and N2 backgrounds, after treatment with GFP RNAi or empty vector. Horizontal bars indicate group means. The difference in log_2_ intensity ratios between GFP RNAi and empty vector is reduced in JU1580 compared to N2 (*p*<0.001, see Methods). (D) *unc-22* dsRNA was administered by injection into the syncytial germline of the mother. 10–14 animals of each genotype were injected and 30 progeny were scored for the twitcher phenotype on each plate. (E) Orsay virus sensitivity of seven wild *C. elegans* isolates representative of the species' diversity. Morphological symptoms were scored 5 d after infection of clean cultures by the Orsay virus filtrate at 23°C. The JU1580 control was performed in duplicate. Bar: standard error on total proportion. *** *p*<0.001.

Since a functional RNAi pathway limits the accumulation of viral RNA in the N2 reference strain, we assessed the exogenous RNAi competency of the bleached culture of JU1580 (JU1580bl) relative to the reference N2 strain. Using external application of dsRNAs by feeding, JU1580bl was found to be highly resistant to RNAi of a somatically expressed gene (*unc-22*) but competent for RNA inactivation of a germline-expressed gene (*pos-1*) (Figure 8A,B). *C. elegans* wild isolates, such as CB4856, were previously known to be variably sensitive to germline RNAi [20]. Here we thus observed for the first time a large variation in sensitivity to somatic RNAi, which does not correlate with germline RNAi sensitivity and thus cannot be due to inability to intake dsRNA from the intestinal lumen. We confirmed insensitivity to somatic RNAi of the JU1580bl isolate using a ubiquitously expressed GFP transgene (*let-858::GFP*), which was inactivated by GFP RNAi in the *C. elegans* N2 reference background, but only modestly repressed in the JU1580bl isolate (Figure 8C, Figure S4). We confirmed that the insensitivity to somatic RNAi also applied when *unc-22* dsRNAs were directly injected into the syncytial germline (Figure 8D). Therefore, the robust accumulation of Orsay virus RNA observed in infected JU1580 may be rendered possible in part by the partial defect in the somatic RNAi pathway of this wild isolate. The accumulation of small RNAs in response to the virus in infected JU1580 indicates, however, that its RNAi response is at least partially active in some tissues, perhaps including the germline.

The germline RNAi competence of JU1580 together with the presence of Orsay virus RNA1 in the somatic gonad raises the possibility that vertical transmission of viral infection could occur in a strain defective for germline RNAi. To examine this possibility, JU1580bl, N2, and *rde-1* were exposed to Orsay virus filtrate. A subset of adult animals from each plate was bleached and their adult offspring collected 4 d later. No evidence for vertical transmission was observed by qRT-PCR for Orsay virus RNA in any strain (Figure S5).

We further tested the efficiency of the RNAi response in six other wild C. *elegans* isolates representative of its worldwide diversity (Figure 8A,B,D). Our results suggest that the somatic RNAi response varies quantitatively in *C. elegans* and is not correlated with germline RNAi sensitivity. Under experimental conditions that yield efficient infection of JU1580bl by Orsay virus, none of the other strains yielded significant levels of morphological symptoms (Figure 8E). Only JU1580bl and JU258 were positive by RT-PCR (unpublished data). Thus, factors other than RNAi competency also contribute to the sensitivity of *C. elegans* to the Orsay virus.

## Discussion

### The First Viruses Infecting *Caenorhabditis*

Here we report the first molecular description, to our knowledge, of viruses that naturally infect nematodes in the wild. The two novel viruses we identified, while clearly related to known nodaviruses, possess unique genomic features absent from all other previously described nodaviruses. These viruses may thus define a novel genus within the family *Nodaviridae* or may even represent prototype species of a new virus family (pending formal classification by the International Committee for the Taxonomy of Viruses). The same range of intestinal symptoms was observed in animals that were infected by the Orsay and Santeuil viruses, further suggesting that these viral infections were causing the cellular symptoms. We observed putative viral particles of the size expected for nodaviruses, and a strong RNA FISH signal in intestinal cells and the somatic gonad of infected animals demonstrating that the virus is present intracellularly. It is likely that further sampling of natural populations of *Caenorhabditis* will yield other viruses of this and other groups. In fact, these symptoms were seen repeatedly in *C. briggsae* animals sampled from different locations in France, and in one instance, a Santeuil virus variant has been identified (unpublished data).

A characteristic feature of these two viruses is the presence of the novel ORF δ. Conservation of sequence length and identity of the ORF δ in these two viruses, and the absence of this ORF in all other described nodaviruses, suggests that this predicted protein is likely to be important for the ability of the virus to infect or replicate in nematodes. Its function is currently unknown, but it is tempting to speculate that this protein may play a role in antagonizing an innate antiviral pathway.

### A Laboratory Viral Infection of a Small Model Animal

The infection of *C. elegans* by the Orsay nodavirus provides an exciting prospect for studies in virology, host cell biology, and antiviral innate immunity. Genetic screens to identify anti-viral factors in model organisms have been limited in large part by the lack of natural infection systems. Although *Drosophila* has been used with great success to examine host-virus interactions for various insect viruses [21] and influenza [1], none of these studies has examined viral infection of the host organism by natural transmission routes. Here we present a novel association between *C. elegans* and a virus that persists in culture through horizontal transmission, causing high damage in intestinal cells yet remarkably little effect on the animal, which continues moving, eating, and producing progeny, although at a lower rate.

De novo infection of naïve animals can be affected by the simple addition of either dead infected animals or homogenized lysates made from infected animals to culture dishes. This is sufficient to seed sustained complete cycles of viral replication, shedding, and infection. With this system, it is now possible to embark on whole genome genetic screens to identify host factors that block any facet of the viral life cycle. Using the current experimental conditions, infection of JU1580bl and *rde-1* mutants in N2 background was highly reproducible. The fact that the reference wild type N2 strain may only sustain a very low yet detectable viral titer makes it a particularly favorable genetic background in which to screen for genes involved in interaction with the virus.

The intestine is a tissue that is particularly exposed to microbes through ingestion, and is a main entry point for pathogens in *C. elegans* as in other animals. In *C. elegans*, the intestinal cells are large and easily amenable to observations by optical microscopy. The viral parasites affect the organization of the polarized epithelial intestinal cells and will likely provide interesting mechanisms and tools to study their cell biology. Clear reorganization occurs in the intermediate filaments that line the apical brush border, as well as in the lipid storage granules, the nuclear membrane, and other intracellular compartments.

The abnormal state of the intestinal cells may slow down progeny production by decreasing the food intake. Alternatively, the presence of viral RNA in the somatic gonad may explain the delay in progeny production, although no gonadal cellular phenotypes have been observed. The presence of viral RNA in the somatic gonad is particularly interesting given the lack of vertical transmission.

### Targeted Mutant Screens with Orsay Virus Confirm a Role for RNAi in Antiviral Defense

Although prior studies have clearly demonstrated a role for *C. elegans* RNAi in counteracting viral infection, these studies utilized either a transgenic system of viral RNA expression [7] or primary culture cells [22],[23]. The observed susceptibility of Orsay virus RNA to RNAi processing in JU1580 animals provides the first evidence in a completely natural setting, without any artificial manipulations, that RNAi serves an antiviral role in nematodes. Coupled to the increase in accumulation of Orsay virus RNA in RNAi pathway mutant strains as compared to wild type N2, these studies demonstrate that the RNAi pathway is an important antiviral defense against Orsay virus. Moreover, these results demonstrate the feasibility of identifying antiviral genes or pathways in this experimental infection system. The mechanism by which the animals prevent transmission to their offspring is unclear, but our initial results with *rde-1* mutants suggest that perturbing germline RNAi is not sufficient to enable vertical transmission.

### Evolution of Viral Sensitivity and Specificity in Natural Populations

The quantitative difference in Orsay nodavirus sensitivity between the N2 and JU1580 wild *C. elegans* genetic backgrounds will allow the identification of a set of host genes that modulate viral sensitivity during evolution of natural host populations. Based on the defect in exogenous RNAi of the JU1580 strain, we speculate that this set will include, but is unlikely to be limited to, genes involved in exogenous RNAi pathways. Support for the role of other genes outside the RNAi pathway comes from our data on natural isolates. Despite the fact that the magnitude of the somatic RNAi defect of the natural isolate PS2025 was comparable to that of JU1580, no evidence of viral RNA accumulation or morphological symptoms was observed following addition of Orsay virus filtrate. Whether PS2025 lacks one or more crucial receptors for viral infection or has alternative antiviral pathways that suppress viral replication is currently unknown.

In addition, the Orsay and Santeuil viruses appear to specifically infect *C. elegans* and *C. briggsae*, respectively. Moreover, the *C. elegans rde-1* mutation in the N2 background confers susceptibility to the Orsay virus, but not to the Santeuil virus (Figure S3C). The two viruses thus provide a system to study host-parasite specificity and its evolution. With the isolation of additional variants of each virus (our unpublished data), viral evolution studies can also be undertaken. Host-parasite evolutionary and ecological interactions can thus be explored at two evolutionary scales, within and between species of both host and parasite. The rapid life cycle of *C. elegans* also allows experimental evolution in the laboratory [24],[25]. This model system, which can include both natural and engineered variants of both virus and host, is thus favorable for combining studies of host-pathogen co-evolution in the laboratory and in natural populations.

## Materials and Methods

### Nematode Field Isolation

*Caenorhabditis* nematodes were isolated on *C. elegans* culture plates seeded with *E. coli* strain OP50 using the procedures described in [10]. JU1264 was isolated from a snail collected on rotting grapes in Santeuil (Val d'Oise, France) on 14 Oct 2007. JU1580 was isolated from a rotting apple sampled in Orsay (Essonne, France) on 6 Oct 2008. When required, cultures were cleared of natural bacterial contamination by frequent passaging of the animals and/or antibiotic treatment (LB plates with 50 µg/ml tetracycline, ampicilline, or kanamycine for 1 h). Infected cultures were kept frozen at −80°C and in liquid N2 as described in [26]. Bleaching was performed as in [26].

### Light Microscopy

When observed with a transillumination dissecting microscope, infected animals displayed a paler intestine than healthy worms. This lack of intestinal coloration occurred all along the entire intestinal tract in *C. briggsae* JU1264 and preferentially in the anterior intestinal tract in *C. elegans* JU1580. Intestinal cells were observed with Nomarski optics with a 63× or 100× objective. The four symptoms used for scoring were 1, the disappearance of gut granules in at least part of a cell; 2, degeneration of the nucleus including a very elongated nuclear or nucleolus (when the rest of the nucleus has degenerated) or the apparent disappearance of the nucleus; 3, the loss of cytoplasmic viscosity visible as a very fluid flow of cytosol within the cell; and 4, the fusion of intestinal cells. Some of these traits may sometimes appear in uninfected animals. We systematically tested for a significant increase after infection of the proportion of animals with symptoms (Fisher's exact test). Note that some of these symptoms can also be caused by microsporidial and bacterial infections. Thus, the diagnostic of a viral infection based on the cellular symptoms requires an otherwise clean culture.

### Live Hoechst 33342 Staining of Nuclei

Animals were washed off a culture plate in 10 ml of ddH_2_0, pelleted and incubated in 10 ml of 10 µg/ml Hoechst 33342 in ddH_2_0 for 45 min with soft agitation, protecting the tube from light with an aluminum foil. The animals were then pelleted and transferred to a new culture plate seeded with *E. coli* OP50. After 2 h, they were mounted and observed with a fluorescence microscope.

### Electron Microscopy

A few adults were washed in 0.2 ml of M9 solution, suspended in 2% paraformaldehyde +0.1% glutaraldehyde, and cut in two on ice under a dissecting microscope for better reagent penetration [27]. Worm pieces were then resuspended overnight in 2% OsO_4_ at 4°C, washed, embedded in 2% low melting point agar, dehydrated in solutions of increasing ethanol concentrations, and embedded in resin (Epon-Araldite). High-pressure freezing was performed using a Leica PACT2 high-pressure freezer [28].

### Progeny Counts

The time course was started by isolating single L4 larvae for *C. elegans* JU1580 and single L3 larvae for *C. briggsae* JU1264. The parent animal then transferred every day to a new plate until the end of progeny production. The plates were incubated at 20°C for 2 d and kept at 4°C until scoring. The few cases where the parent died before the end of its laying period were not included. Some progeny died as embryos in both infected and non-infected cultures (non-significant effect of treatment; unpublished data). The timing of progeny production was analyzed in R using a Generalized Linear Model using infection status, day, individual (nested in infection status), and Infection Status×Day as explanatory variables, assuming a Poisson response variable and a log link function. Individual, day and Infection Status×Day were the significant explanatory variables for both JU1264 and JU1580 (*p*<0.001).

### Infectious Filtrate Preparation and Animal Infections

Nematodes were grown on 10 plates (90 mm diameter) until just starved, resuspended in 15 ml of 20 mM Tris-Cl pH 7.8, and pelleted by low-speed centrifugation (5,000 g). The supernatant was centrifuged twice at 21,000 g for 5 min (4°C) and pellets discarded. The supernatant was passed on a 0.2 µm filter. 55 mm culture plates were prepared with 2–5 young adults of N2, *rde-1(ne219)*, or JU1580bl. At the same time (Figures 1K, 4A,B, 5, 7B, and S3), or the following day (Figures 5C, 7A), 30 µl of infectious filtrate was pipetted onto the bacterial lawn. The cultures were incubated at 20°C except otherwise indicated. When both *C. elegans* and *C. briggsae* were grown in parallel, an incubation temperature of 23°C (indicated in the figure legends) was used so that both species developed at similar speeds. Maintenance over more than 4 d after re-infection was performed by transferring a piece of agar (approx. 0.1 cm^3^) every 2–3 d to a new plate with food.

### High-Throughput Sequencing

Phenol-chloroform purified DNA and RNA from infected JU1580 and JU1264 animals were subject to random PCR amplification as described [29]. The amplicons were then pyrosequenced following standard library construction on a Roche Titanium Genome Sequencer. Raw sequence reads were filtered for quality and repetitive sequences. BLASTn and BLASTx were used to identify sequences with limited similarity to known viruses in Genbank. Contigs were assembled using the Newbler assembler. To confirm the assembly, primers for RT-PCR were designed to amplify overlapping fragments of ∼1.5 kb. Amplicons were cloned and sequenced.

### 5′ and 3′ RACE

5′ RACE was performed according to standard protocols (Invitrogen 5′ RACE kit). 3′ RACE was performed by first adding a polyA tail using PolyA polymerase (Ambion) and then using Qiagen 1-step RT-PCR kit with gene specific primers and an oligo-dT-adapter primer. Products were cloned into pCR4 and sequenced using standard Sanger chemistry.

### Small RNA Sequencing

4–6 90 mm plates with 15–20 adults (JU1580 or bleached JU1580) were grown for 4 d at 20°C. Mixed stage animals from all plates were collected, pooled, and frozen at −80°C. Total RNA was extracted using the mirVana miRNA isolation kit (Ambion). Small RNAs were size selected to 18–30 bases by denaturing polyacrylamide gel fractionation. A cDNA library that did not depend on 5′-monophosphates was constructed by tobacco acid pyrophosphatase treatment using adapters recommended for Solexa sequencing as described previously [30]. Each sample was labeled with a unique four base pair barcode. cDNA was purified using the NucleoSpin Extract II kit (Macherey & Nagel). Small RNA libraries were sequenced using the Illumina/Solexa GA2 platform (Illumina, Inc., San Diego, CA). Fastq data files were processed using custom Perl scripts. Reads with missing bases or whose first four bases did not match any of the expected barcodes were excluded. Reads were trimmed by removing the first four nucleotides and any 3′ As. The obtained inserts were collapsed to unique sequences, retaining the number of reads for each sequence. Sequences in the expected size range (18–30 nucleotides) were aligned to the *C. elegans* genome (WS190) downloaded from the UCSC Genome Browser website (http://genome.ucsc.edu/) [31] and the JU1580 partial virus genome using the ELAND module within the Illumina Genome Analyzer Pipeline Software, v0.3.0. Figure 6 is based on unique sequences (multiple reads of the same sequence were collapsed) with perfect and unambiguous alignment to the Orsay virus genome. Small RNA sequence data were submitted to the Gene Expression Omnibus under accession number GSE21736.

### Neighbor-Joining Phylogenetic Analysis

The predicted amino acid sequences from Orsay and Santeuil nodaviruses were aligned using ClustalW to the protein sequences of the following nodaviruses. Capsid Protein: Barfin1 flounder nervous necrosis virus NC_013459, Barfin2 flounder virus BF93Hok RNA2 NC_011064, Black beetle virus NC_002037, Boolarra virus NC_004145, *Epinephelus tauvina* nervous necrosis virus NC_004136, Flock house virus NC_004144, *Macrobrachium rosenbergii* nodavirus RNA-2 NC_005095, Nodamura virus RNA2 NC_002691, Pariacoto virus RNA2 NC_003692, Redspotted grouper nervous necrosis virus NC_008041, Striped Jack nervous necrosis virus RNA2 NC_003449, Tiger puffer nervous necrosis virus NC_013461, Wuhan nodavirus ABB71128.1, and American nodavirus ACU32796.1. 1,000 bootstrap replicates were performed.

RNA Polymerase: Barfin flounder nervous necrosis virus YP_003288756.1, Barfin flounder virus BF93Hok YP_002019751.1, Black beetle virus YP_053043, Boolarra virus NP_689439, *Epinephelus tauvina* nervous necrosis virus NP_689433.1, Flock house virus NP_689444.1, Nodamura virus NP_077730, Pariacoto virus NP_620109.1, Redspotted grouper nervous necrosis virus YP_611155.1, Striped Jack nervous necrosis virus NP_599247.1, Tiger puffer nervous necrosis virus YP_003288759.1, *Macrobrachium rosenbergii* nodavirus NP_919036.1, Wuhan_Nodavirus AAY27743, and American nodavirus SW-2009a ACU32794.1. 1,000 bootstrap replicates were performed.

### RT-PCR

Nematodes from two culture plates were resuspended in M9 and then washed three times in 10 ml M9. RNA was extracted using Trizol (Invitrogen) (5–10 vol∶vol of pelleted worms) and resuspended in 20 µl in RNAse-free ddH_2_O. 5 µg of RNA were reverse transcribed using SuperscriptIII (Invitrogen) in a 20 µl volume. 5 µl were used for PCR in a 20 µl volume (annealing temperature 60°C, 35 cycles). For the Orsay nodavirus, the reverse transcription used the GW195 primer (5′ GACGCTTCCAAGATTGGTATTGGT) and the PCR oTB3 (5′ CGGATTCTCGACATAGTCG) and oTB4 (5′GTAGGCGAGGAAGGAGATG). For the Santeuil nodavirus, reverse transcription used oTB6RT (5′ GGTTCTGGTGGTGATGGTG) and PCR oTB5 (5′ GCGGATGTTCTTCACGGAC) and oTB6 (5′ GTCAGTAGCGGACCAGATG).

### One-Step RT-PCR

Animals from one 55 mm culture plate plus viral filtrate (see infection procedure) were washed twice in M9. RNA was extracted using 1 ml Trizol (Invitrogen) and resuspended in 10 µl DEPC-treated H_2_O. 0.1 µl was used for RT-PCR using the OneStep RT-PCR Kit (Qiagen). Primers annealed to viral RNA1 (GW194 and GW195).

### qRT-PCR

cDNA was generated from 1 µg total RNA with random primers using Superscript III (Invitrogen). cDNA was diluted to 1∶100 for qRT-PCR analysis. qRT-PCR was performed using either QuantiTect SYBR Green PCR (Qiagen) or ABsolute Blue SYBR Green ROX (Thermo Scientific). The amplification was performed on a 7300 Real Time PCR System (Applied Biosystems). Each sample was normalized to *ama-1*, and then viral RNA1 (primers GW194: 5′ ACC TCA CAA CTG CCA TCT ACA and GW195: 5′ GAC GCT TCC AAG ATT GGT ATT GGT) levels were compared to those present in re-infected bleached JU1580 animals.

### Northern Blotting

For Northern blots, 0.5 µg of total RNA extracted from JU1264 and JU1264bl animals were electrophoresed through 1.0% denaturing formaldehyde-MOPS agarose gels. RNA was transferred to Hybond nylon membranes and then subject to UV cross-linking followed by baking at 75°C for 20 min. Double stranded DNA probes targeting the RNA1 segment of Santeuil nodavirus (nt 1141–1634) and the RNA2 segment of Santeuil nodavirus (nt 1833–2308) were generated by random priming in the presence of α-^32^P dATP using the Decaprime kit (Ambion). Blots were hybridized for 4 h at 65°C in Rapid hyb buffer (GE Healthcare) and washed in 2XSSC/0.1%SDS 5 min×2 at 25°C, 1XSSC/0.1%SDS 10 min×2 at 25°C, 0.1XSSC/0.1%SDS 5 min×4 at 25°C, and 0.1XSSC/0.1%SDS 15 min×2 at 42°C and 0.1XSSC/0.1%SDS 15 min×1 at 68°C. For strand specific riboprobes, ^32^P labeled RNA was generated by in vitro transcription with either T7 or T3 RNA polymerase (Ambion) in the presence of α-^32^P UTP. The target plasmid contained a cloned region of the Santeuil nodavirus RNA1 segment (nt 523–1022) and was linearized with either PmeI or NotI, respectively. For the riboprobes, blots were hybridized at 70°C and then sequentially washed as follows: 2XSSC/0.1%SDS 5 min×2 at 68°C, 1XSSC/0.1%SDS 10 min×2 at 68°C, 0.1XSSC/0.1%SDS 10 min×2 at 68°C, and 0.1XSSC/0.1%SDS 20 min×1 at 73°C. The Santeuil RNA1 segment migrates at approximately the same position as the 28S ribosomal RNA. Under the extended exposure time (72 h) needed to visualize the negative sense genome, low levels of non-specific binding to the 28S RNA become apparent (Figure 4D).

### RNA Interference

For *pos-1* and *unc-22* RNAi using bacteria as the dsRNA source, bacterial clones from the Ahringer library expressing dsRNAs [32] (available through MRC Geneservice) were used to feed *C. elegans* on agar plates. For the *pos-1* experiment, bacteria were concentrated 10-fold by centrifugation prior to seeding the plates. A *C. briggsae Cbr-lin-12* fragment [33] was used as a negative control as it does not match any sequence in *C. elegans*. Three or four L4s were deposited on an RNAi plate, singly transferred the next day to a second RNAi plate, and their progeny scored after 2 d (*pos-1*) or 3 d (*unc-22*) at 23°C.

For *unc-22* dsRNA synthesis and injection, the *unc-22* fragment in the Ahringer library clone was amplified by PCR using the T7 primer and in vitro transcribed with the T7 polymerase using the Ambion MEGAscript kit, according to the manufacturer's protocol [34]. *Cel-unc-22* dsRNAs were injected at 50 ng/µl into both gonadal arms of young hermaphrodite adults of the relevant strain. The animals were incubated at 20°C. The adults were transferred to a new plate individually on the next day, and the proportion of twitching progeny scored 3 d later, touching each animal with a platinum-wire pick to induce movement.

For GFP RNAi, transgenic N2 and JU1580 strains were generated expressing the ubiquitously expressed *let-858::GFP* and the pharyngeal marker *myo-2::DsRed* as an extrachromosomal array. Bacteria expressing dsRNA against GFP cDNA were used to feed animals on agar plates. An empty vector was used as a negative control. Two or three L4s were deposited on a 55 mm RNAi plate, grown at 20°C for 3 d, and the GFP/DsRed expression levels in their offspring measured using flow cytometry (Union Biometrica) as described previously [35]. Offspring from two RNAi plates were combined for sorting. Each combination of RNAi vector and strain was repeated in at least triplicate. GFP and DsRed intensities were obtained from 14 wormsorter runs including 3–4 replicate runs for N2 and JU1580 after treatment with GFP RNAi or empty vector. A larger proportion of N2 animals showed reporter expression compared to JU1580 animals (Figure S4, top). To control for this difference between strains, animals with no reporter expression were excluded by requiring DsRed intensities to exceed a cutoff set to the median 99th percentile from three control runs of animals with no array present (Figure S4). A linear regression model was fitted to the median log2(GFP/DsRed) intensity ratios including strain, treatment, and an interaction term as explanatory variables. The interaction term was significantly different from zero at *p*<0.001.

### RNA Fluorescent In Situ Hybridization (FISH)

A segment of Orsay virus RNA1 was generated with primers GW194 and GW195 and cloned into pGEM-T Easy (Promega). Fluorescein labeled probe was generated from linearized plasmid using the Fluorescein RNA Labeling Mix (Roche) and MEGAscript SP6 transcription (Ambion). JU1580bl animals were infected with Orsay virus filtrate and grown for 4 d at 20°C on 90 mm plates. Control animals were grown under the same conditions in the absence of virus. In situ hybridization was performed essentially as previously described [36]. The fluorescent RNA probe was visualized directly on an Olympus FV1000 Upright microscope.

Genbank Sequences: Accession numbers for Orsay and Santeuil virus contigs: HM030970–HM030973. Small RNA sequencing data at GEO: GSE21736.

## Supporting Information

Figure S1 **Putative viral particles in transmission electron microscopy of intestinal cells of infected** ***C. elegans*** **JU1580 adult hermaphrodites.** On the right are shown higher magnifications of the parts delimited by a black rectangle in the left micrograph, showing putative viral particles (arrows). (A) Putative viral particles are found in an intracellular multi-membrane compartment. The particles in the upper left of the inset of (A) are ribosomes; the putative viral particles in the lower two-thirds are characterized by a slightly larger and more regular ring appearance (ca. 20 nm diameter). (B) Putative viral particles similar to those in Figure 2H are visible in the intestinal lumen, close to microvilli. These particles are clearly larger and distinct from the ribosomes (r) seen on the lower left of the inset. The animals were fixed using conventional fixation in (A) and high-pressure freezing in (B).
(3.41 MB PPT)

Figure S2 **Infection does not alter brood size, but results in delayed progeny production in** ***C. briggsae*** **JU1264.** (A,B) Boxplots of the distribution of brood size in naturally infected and bleached (“bl”) cultures of *C. elegans* JU1580 (A) and *C. briggsae* JU1264 (B) at 20°C. The line indicates the median, the box the lower and upper quartiles, and the whiskers the 10^th^ and 90^th^ percentiles. Brood size is not significantly different in infected versus non-infected cultures (Wilcoxon test *p* = 1.0 for JU1264, *p* = 0.81 for JU1580). (C,D) Progeny number over time in JU1580 (C) and JU1264 (D). Infection results in a significant change in the timing of progeny production (Generalized linear model, Treatment×Day: *p*<0.001 in both cases). Note that time 0 corresponds to the L4 stage in the experiment in (A,C) and the L3 stage in (B,D).
(0.33 MB PDF)

Figure S3 **Scoring of morphological symptoms after exposure of various wild isolates and** ***rde-1*** **mutants.** (A) Specificity of infection by the Orsay nodavirus. Each *Caenorhabditis* strain was mock-infected (−) or infected with a virus filtrate (+). The proportion of worms with morphological infection symptoms after 7 d at 23°C is shown for the same experiment as in Figure 5A. (B) Specificity of infection by the Santeuil nodavirus. The proportion of worms with morphological infection symptoms after 4 d at 23°C is shown for the same experiment as in Figure 5B. (C) Santeuil virus sensitivity of *rde-1* mutants in the *C. elegans* N2 background. Morphological symptoms were scored 5 d after infection at 23°C by the Santeuil virus filtrate. * *p*<0.05; *** *p*<0.001.
(0.29 MB PDF)

Figure S4 **Quantification of GFP transgene expression and silencing.** (Top) Reporter genes are expressed in a larger proportion of animals from N2 compared to JU1580. Shown are the number of animals according to binned log_2_ DsRed intensities. Each line corresponds to one flow cytometry run with colors indicating strain and treatment as explained in the color legend. The extrachromosomal array was inherited more efficiently in N2 than in JU1580, making it necessary to analyze only those animals carrying the array. (Bottom) Each data point corresponds to the difference between treatment with GFP RNAi and empty vector in N2 (blue) and JU1580 (red) observed for animals with log_2_ DsRed intensity in a given bin. Dots indicate the difference between the means of median log_2_(GFP/DsRed) ratios for treatment with GFP RNAi and empty vector. Vertical bars indicate standard errors. The cutoff for DsRed intensities is indicated in both panels by a vertical dotted line.
(0.29 MB AI)

Figure S5 **Quantitative analysis of vertical transmission of Orsay virus.** N2, JU1580bl, and *rde-1* (5 replicates each) were infected with Orsay virus. After 4 d 25 adults from each plate were bleached onto a new, uninfected plate. The remaining adults were collected for RNA extraction. The offspring from the bleached adults were collected for RNA extraction after 4 d. As a control, virus was added to plates and incubated in the absence of animals for 4 d. Viral RNA levels were determined by qRT-PCR and normalized to *gapdh*. Viral RNA level is shown on a log scale, using a reference value of 1 for the infection of JU1580.
(0.27 MB AI)
