# Supplementary figures and images for: Natural and Experimental Infection of Caenorhabditis Nematodes by Novel Viruses Related to Nodaviruses
Source: PLoS Biol. 2011 Jan 25;9(1):e1000586. doi: 10.1371/journal.pbio.1000586 (PMC3026760; doi:10.1371/journal.pbio.1000586)

## Slide 1
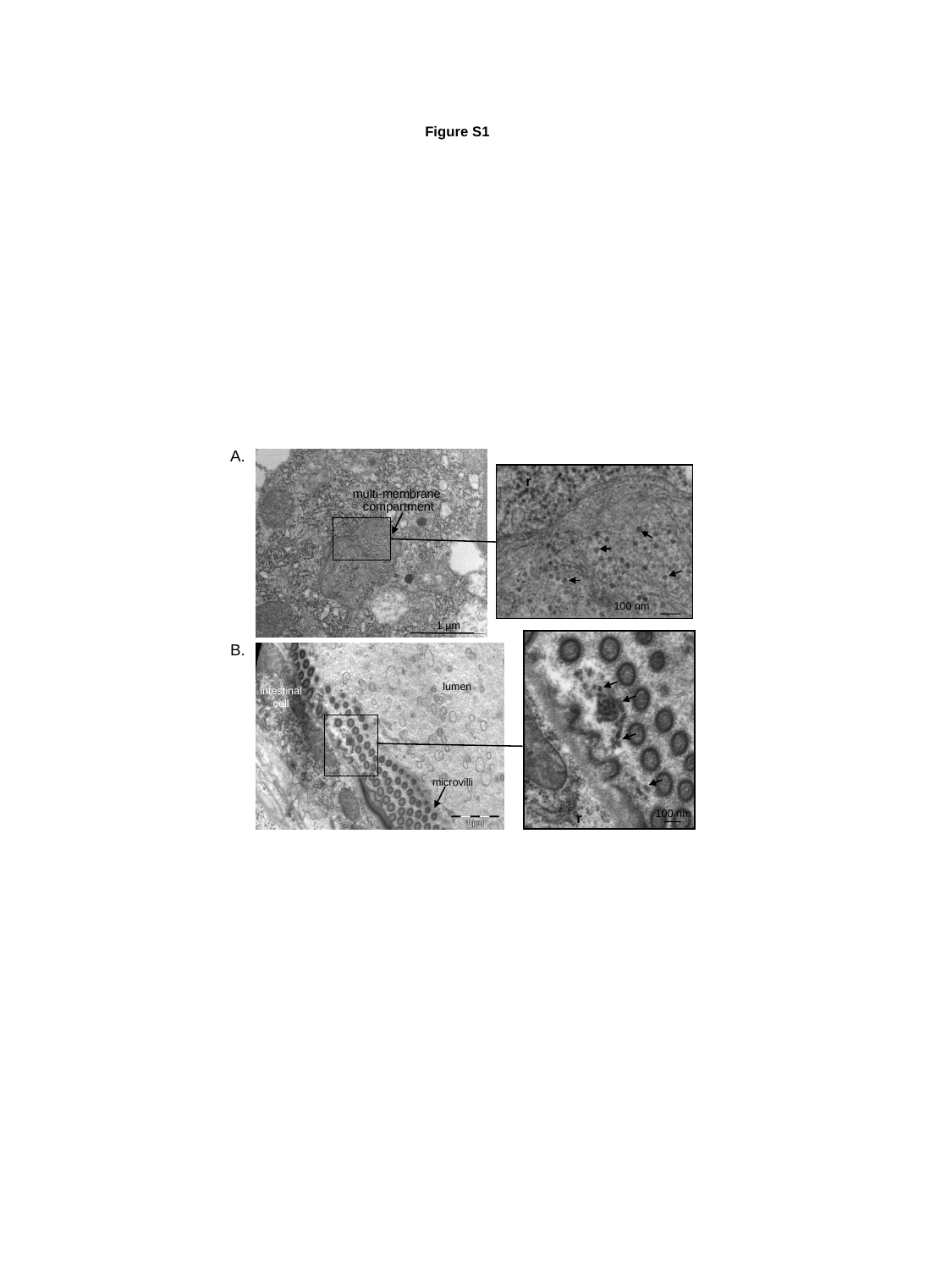

Figure S1
A.
multi-membrane
compartment
1 µm
r
100 nm
B.
lumen
intestinal
cell
microvilli
100 nm
r

Supplement: Figure S1 — Putative viral particles in transmission electron microscopy of intestinal cells of infected C. elegans JU1580 adult hermaphrodites. On the right are shown higher magnifications of the parts delimited by a black rectangle in the left micrograph, showing putative viral particles (arrows). (A) Putative viral particles are found in an intracellular multi-membrane compartment. The particles in the upper left of the inset of (A) are ribosomes; the putative viral particles in the lower two-thirds are characterized by a slightly larger and more regular ring appearance (ca. 20 nm diameter). (B) Putative viral particles similar to those in Figure 2H are visible in the intestinal lumen, close to microvilli. These particles are clearly larger and distinct from the ribosomes (r) seen on the lower left of the inset. The animals were fixed using conventional fixation in (A) and high-pressure freezing in (B). (3.41 MB PPT) [file pbio.1000586.s001.ppt]

Figure S2

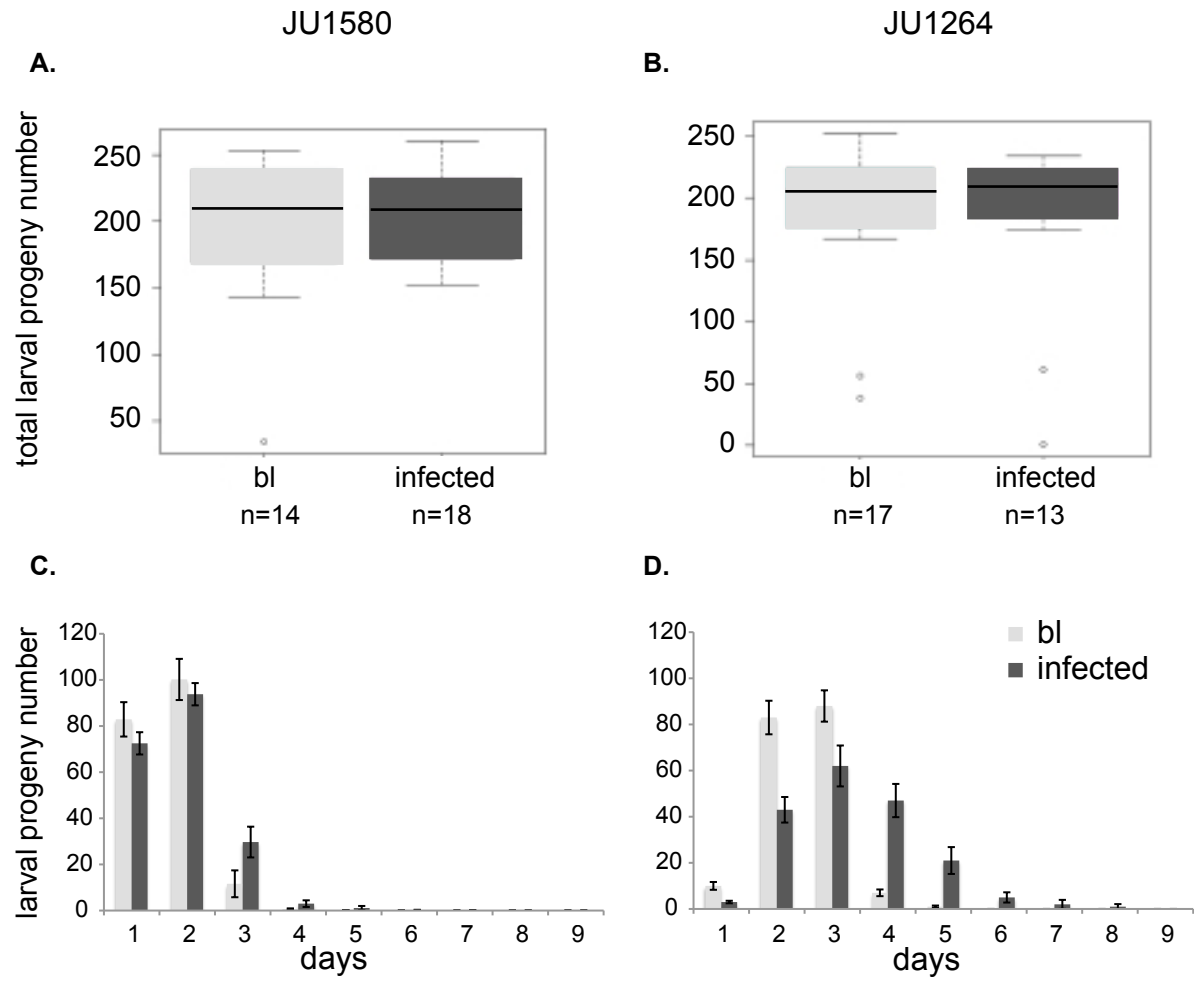

Supplement: Figure S2 — Infection does not alter brood size, but results in delayed progeny production in C. briggsae JU1264. (A,B) Boxplots of the distribution of brood size in naturally infected and bleached (“bl”) cultures of C. elegans JU1580 (A) and C. briggsae JU1264 (B) at 20°C. The line indicates the median, the box the lower and upper quartiles, and the whiskers the 10th and 90th percentiles. Brood size is not significantly different in infected versus non-infected cultures (Wilcoxon test p = 1.0 for JU1264, p = 0.81 for JU1580). (C,D) Progeny number over time in JU1580 (C) and JU1264 (D). Infection results in a significant change in the timing of progeny production (Generalized linear model, Treatment×Day: p<0.001 in both cases). Note that time 0 corresponds to the L4 stage in the experiment in (A,C) and the L3 stage in (B,D). (0.33 MB PDF) [file pbio.1000586.s002.pdf]

Figure S3

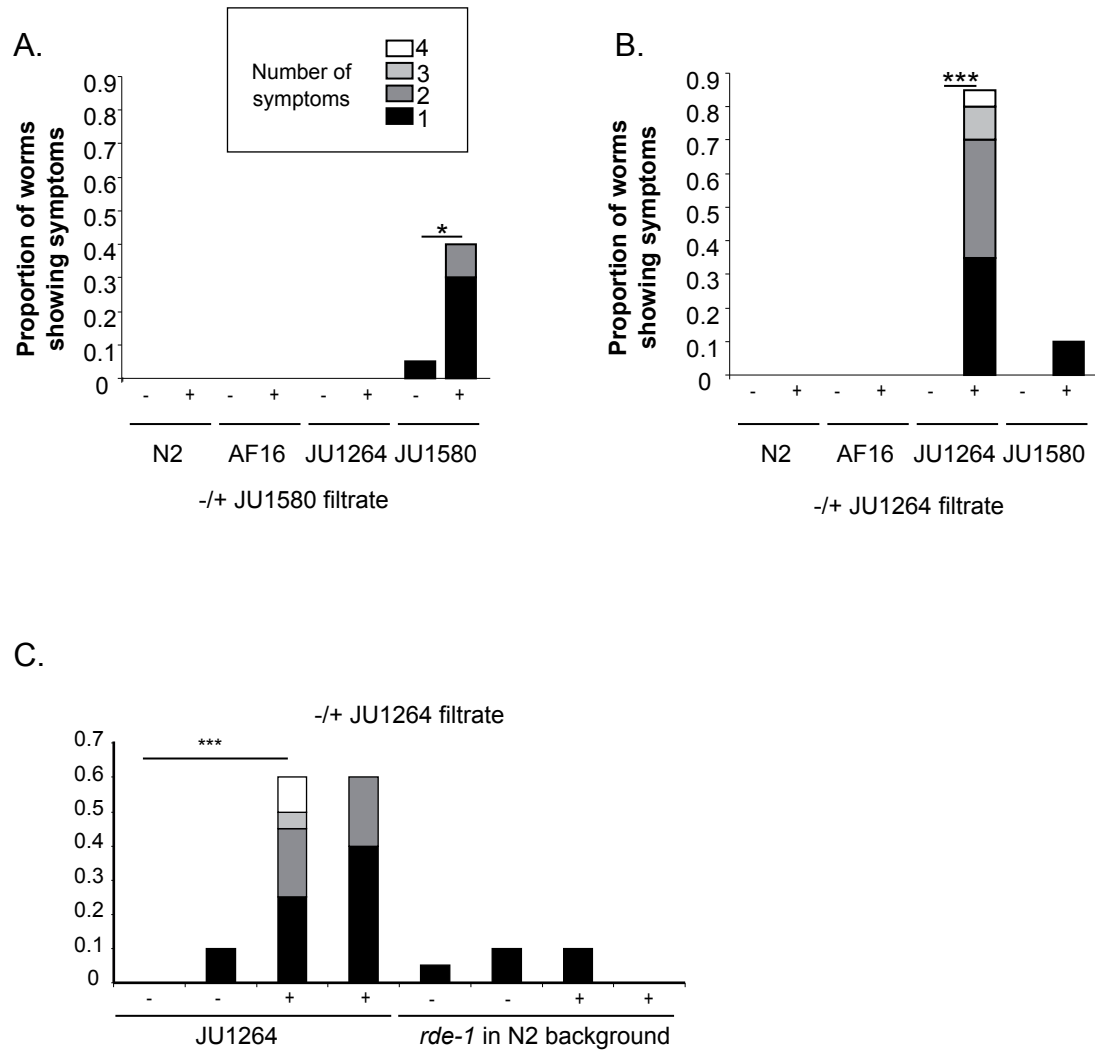

Supplement: Figure S3 — Scoring of morphological symptoms after exposure of various wild isolates and rde-1 mutants. (A) Specificity of infection by the Orsay nodavirus. Each Caenorhabditis strain was mock-infected (−) or infected with a virus filtrate (+). The proportion of worms with morphological infection symptoms after 7 d at 23°C is shown for the same experiment as in Figure 5A. (B) Specificity of infection by the Santeuil nodavirus. The proportion of worms with morphological infection symptoms after 4 d at 23°C is shown for the same experiment as in Figure 5B. (C) Santeuil virus sensitivity of rde-1 mutants in the C. elegans N2 background. Morphological symptoms were scored 5 d after infection at 23°C by the Santeuil virus filtrate. * p<0.05; *** p<0.001. (0.29 MB PDF) [file pbio.1000586.s003.pdf]
